# Supplementary material for: Functional Analysis of the Major Pilin Proteins of Type IV Pili in Streptococcus sanguinis CGMH010
Source: Int J Mol Sci. 2024 May 15;25(10):5402. doi: 10.3390/ijms25105402 (PMC11121087; doi:10.3390/ijms25105402)
Supplement: Supplementary file 1 [file ijms-25-05402-s001.zip › ijms-2963946-supplementary.pdf]

Table S1. Primers used in this study.

**Primer**	**Sequences**[c]	**Purpose**
PilA1-BamHI-S	CGCGGGATCCGCGATTCCAGCTATTACTTC	Construction of pET28a/*pilA1*
PilA1-XhoI-AS	CGCGCTCGAGAGTTCTTATTAACCCCGTTTG	
PilA2-BamHI-S	CGCGGGATCCGCGGTTCCGGCTCTCACTTC	Construction of pET28a/*pilA2*
PilA2-XhoI-AS	CGCGCTCGAGAGTTGGTATTTGAACCACTAT	
PilA3-BamHI-S	CGCGGGATCCGCGATTCCAGCTATCACTTC	Construction of pET28a/*pilA3*
PilA3-XhoI-AS	CGCGCTCGAGAATTTTTATTAACTCCATTTG	
PilA1-501-S[a]	TGGTGCAGGCCGGTGAAAAG	Construction of strain Δ*pilA1*
PilA1-1506-BamHI-AS[a]	CGCGGGATCCTAACATTTTGAATAGATCTCCTATT	
PilA1-1936-XbaI-S	CGCGTCTAGAAAGAACTAACGCAAATCATCAAATC	
PilA1-2944-AS	GGGTTGTACCACTAATAGTATGTGC	
PilA2-1052-S	CGTCTGACAGGGATGATTAC	Construction of strain Δ*pilA2*
PilA2-2057-BamHI-AS	CGCGGGATCCTCGCACTAACTTCTCCTAATTC	
PilA2-2502-XbaI-S[a]	CGCGTCTAGAACCAACTAGCTAAATGTAGTTTAAA	
PilA2-3507-AS[a]	CCGTCAAACTTTCCTGTCAT	
PilA3-1587-S[b]	CATTATCGCTATCATTGCAGCTGTA	Construction of strain Δ*pilA3*
PilA3-2589-BamHI-AS	CGCGGGATCCTAACATTTAAATATTCTCCTATTTT	
PilA3-3022-XbaI-S[a]	CGCGTCTAGAAAAAAATTAATAGACTGCAGAATTAA	
PilA3-4024-AS[a,b]	GTTCCTAACTCACTAACATTCCAGT	
NP-erm-BamHI-S	AATGGATCCTTAAGAAGGAGTGATTACATG	Amplification of *erm*
NP-erm-XbaI-AS	CGATCTAGACTATTATTTCCTCCCGTTAAA	
PilA3-2589-NdeI-AS	CGCGCATATGTAACATTTAAATATTCTCCTATTTT-	Construction of strain Δ*pilA13*
Non-Km-XbaI-AS	TTTCTAGAGTACTAAAACAATTCATCCAGTAAAA-	
Non-polar-Km-S-NdeI	TTCATATGGGAAGGAAATAATAAATGGCTAAAATG-	

[a], also used in the construction of Δ*pilA12* and Δ*pilA123*

[b], also used in the construction of Δ*pilA13*

[c], Restriction endonuclease recognition sequences are underlined.

Figure S1

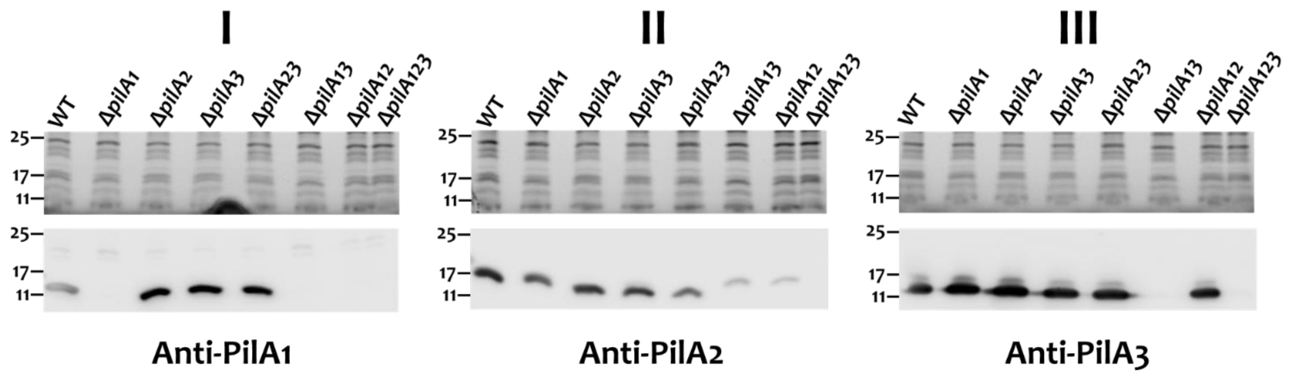

Figure S1.The expression of PilA proteins in *S. sanguinis* CGMH010 and its *pilA*-deletion derivatives. 20 μg of total cell lysate from each strain was separated on 12% SDS-PAGE gels prepared using the TGX Stain-Free system (top images) and analyzed by western analysis (bottom images). The strains are indicated above the gel images. Antisera used for each blot are listed below the blot. The molecular weight in kDa is shown to the left of both the gel and the blot. WT, wild-type *S. sanguinis* CGMH010.
